# Selection of diverse strains to assess broad coverage of the bivalent FHbp meningococcal B vaccine

**DOI:** 10.1038/s41541-019-0154-0

**Published:** 2020-01-29

**Authors:** Shannon L. Harris, Cuiwen Tan, John Perez, David Radley, Kathrin U. Jansen, Annaliesa S. Anderson, Thomas R. Jones

**Affiliations:** 1 Pfizer Vaccine Research and Development, Pearl River, NY USA; 2 Pfizer Vaccine Research and Development, Collegeville, PA USA

**Keywords:** Vaccines, Meningitis

## Abstract

MenB-FHbp is a recombinant meningococcal serogroup B (MenB) vaccine composed of 2 factor H binding proteins (FHbps). Meningococcal vaccines targeting polysaccharide serogroup A, C, Y, and W capsules were licensed upon confirmation of bactericidal antibody induction after initial efficacy studies with serogroup A and C vaccines. Unlike meningococcal polysaccharide vaccines, wherein single strains demonstrated bactericidal antibodies per serogroup for each vaccine, MenB-FHbp required a more robust approach to demonstrate that bactericidal antibody induction could kill strains with diverse FHbp sequences. Serum bactericidal assays using human complement were developed for 14 MenB strains, representing breadth of meningococcal FHbp diversity of ~80% of circulating MenB strains. This work represents an innovative approach to license a non-toxin protein vaccine with 2 antigens representing a single virulence factor by an immune correlate, and uniquely demonstrates that such a vaccine provides coverage across bacterial strains by inducing broadly protective antibodies.

## Meningococcal B vaccine: measuring efficacy

*Neisseria meningitidis* is an important cause of invasive meningococcal disease, effective vaccines exist for some serogroups but immunogenicity to the MenB group is poor. Thomas R. Jones and colleagues examine serum bactericidal responses from volunteers challenged with MenB-FHbp – a recombinant MenB vaccine containing two Factor H (FH)-binding proteins. Serum bactericidal responses are tested against 14 MenB clinical isolates selected in an unbiased manner to cover the vast breadth of FHbp antigen and epidemiological diversity. This work demonstrates the broad efficacy of the MenB-FHbp vaccine using a serum bactericidal activity as a surrogate of protection.

## Introduction

Although transmission of *Neisseria meningitidis* usually results in asymptomatic colonization of the upper respiratory tract, in some individuals, bacteremia and invasive meningococcal disease (IMD) occur.^1–3^ IMD commonly presents as meningitis and/or septicemia; pneumonia, septic arthritis, epiglottitis, and otitis media are less frequently observed.^4,5^ A high case fatality rate is associated with IMD (10–15%),^6,7^ and ~20% of survivors have serious life-long sequelae such as limb amputation, hearing loss, and neurologic impairment.^8,9^

Nearly all meningococcal disease worldwide is caused by 6 of the 12 characterized meningococcal serogroups (ie, A, B, C, W, X, and Y).^10–12^ Effective vaccines based on capsular polysaccharides have been developed for serogroups A, C, W, and Y.^2^ However, immunogenicity of the MenB polysaccharide is poor because of similarity to polysialic acid structures present on human neuronal cells.^13,14^ During recent years, meningococcal serogroup B (MenB) in particular has been associated with a large proportion of IMD in Europe,^15^ the United States,^16^ Canada,^17^ Australia,^18^ and New Zealand.^19^ Although vaccines based on outer membrane vesicles (OMVs) have been successfully used to control epidemics caused by a single MenB outbreak strain,^20,21^ the generated immune response is predominantly against the highly variable porin A protein (PorA).^22–25^ Therefore, effectiveness is generally limited to the target strain. Consequently, surface-exposed proteins capable of inducing protective bactericidal antibodies across diverse MenB strains have been sought for the development of a broadly effective MenB vaccine.

**Fig. 1:**
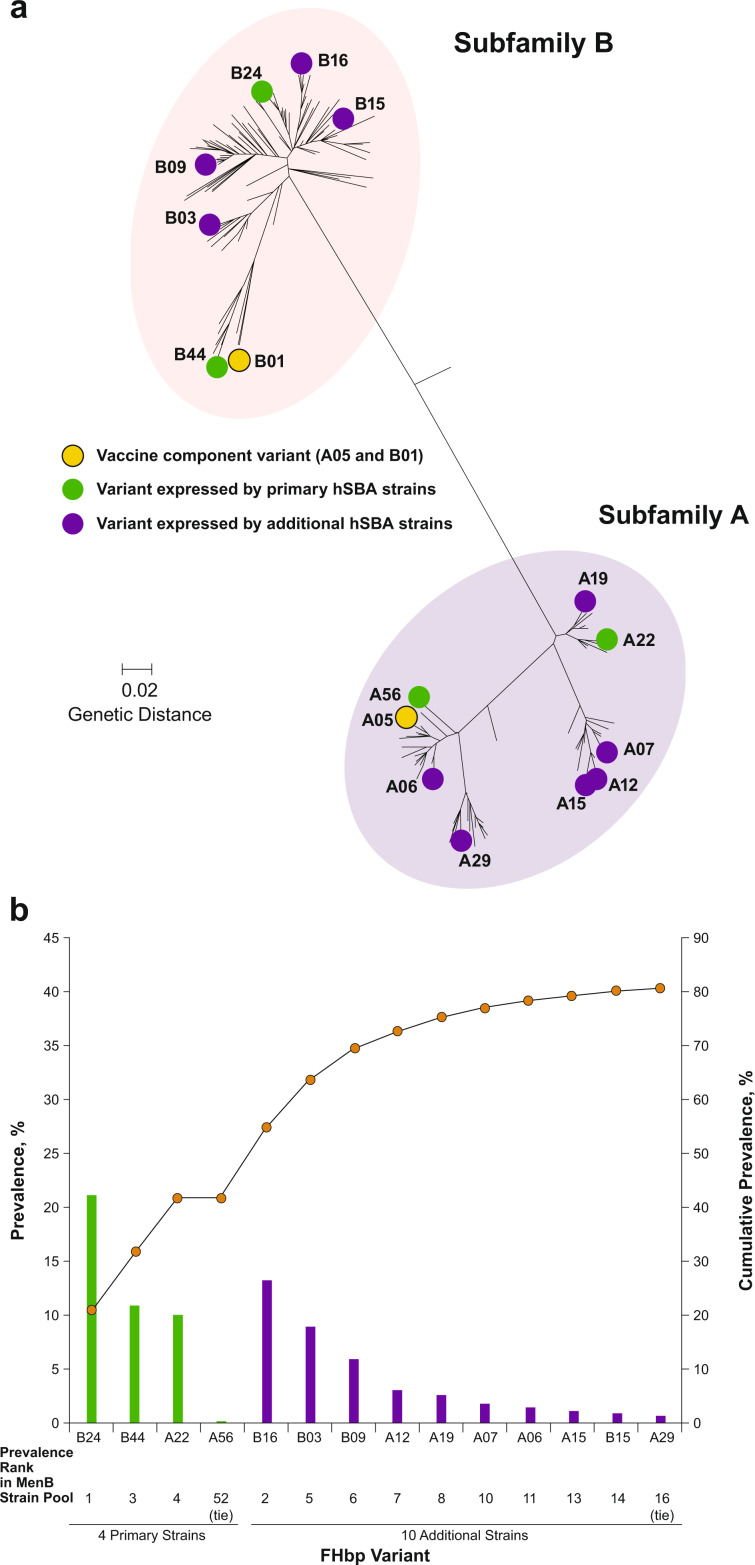
Factor H binding protein (FHbp) phylogenetic tree: Primary and additional *Neisseria meningitidis* serogroup B (MenB) test strain variants^32^ and variant prevalence of primary and additional MenB test strains. In **a**, the phylogenetic and FHbp subfamily relationship of the FHbp variants expressed by the four primary and 10 additional MenB test strains is illustrated. The scale bar indicates genetic distance based on protein sequence. The amino acid sequence identity within FHbp subfamilies is ≥83%.^33^ hSBA = serum bactericidal assay using human complement. Adapted from Ostergaard, L. et al. A bivalent meningococcal B vaccine in adolescents and young adults. N. Engl. J. Med. 377, 2349–2362 (2017). In **b**, variant prevalence (left vertical axis; bars) and cumulative prevalence (right vertical axis; circles) are based on the MenB isolate collection (*n* = 1263). Variants are ordered based on their prevalence rank in the MenB SBA isolate collection. Note that scales are different between left and right *y*-axes. SBA serum bactericidal assay.

Factor H binding protein (FHbp; also known as LP2086 and GNA1870), a conserved surface-exposed lipoprotein expressed on nearly all strains of MenB, was identified as such a target.^26–31^ Based on amino acid sequence, FHbp variants segregate into 2 immunologically distinct subfamilies (termed subfamily A and subfamily B); each MenB strain expresses a single subfamily variant (Fig. 1a).^32,33^

MenB-FHbp (Trumenba®, bivalent rLP2086; Pfizer Inc, Philadelphia, PA, USA) is a bivalent, recombinant protein MenB vaccine composed of equal amounts of 2 recombinant lipidated FHbp antigens, one from subfamily A (variant A05) and the other from subfamily B (variant B01).^34^ Importantly, it is predicted that this combination of FHbp variants is capable of providing protection against diverse MenB strains.^33,35^ MenB-FHbp has been approved for the prevention of IMD in several countries and regions, including the United States, Canada, Europe, and Australia.^34,36–38^ Another MenB vaccine, MenB-4C (Bexsero®, 4 CMenB; GlaxoSmithKline Vaccines, Srl, Siena, Italy), also has a recombinant FHbp component (nonlipidated variant 1.1 from subfamily B) as well as 2 other recombinant protein antigens and an OMV.^39^ Thus, MenB-4C is different from MenB-FHbp, which contains two variants of a single antigen to afford broad coverage.^40^

The serum bactericidal assay using human complement (hSBA) measures complement-dependent, antibody-mediated lysis of meningococcal bacteria. An hSBA titer is defined as the highest serum dilution killing ≥50% of assay bacteria;^41^ an hSBA titer ≥1:4 is the accepted correlate of protection against meningococcal disease,^41–43^ and hSBA response rates based on this correlate have been used as surrogates for meningococcal vaccine efficacy.^43^ The SBA response rate has been specifically correlated with natural protection for the serogroup C and A polysaccharide vaccines.^41^ Because serogroup-specific polysaccharides are not variable, a single strain from each serogroup was sufficient to infer broad vaccine coverage. MenB OMV vaccines are also efficacious and vaccine-elicited hSBA titers correlated with protection against the target strain causing the epidemic.^44–46^ Accurately predicting strain coverage of protein-based vaccines is more complex using hSBA than for vaccines targeting capsular polysaccharides, given that protein sequence diversity and variability in expression levels differ among the different meningococcal disease strains.^47^ For example, PorA is the predominant target for serum bactericidal antibodies conferring protection after OMV vaccine immunization.^48,49^ PorA is a cell surface porin whose small cell surface exposed region has a high degree of sequence diversity. It has been estimated that protective immunity would need to be demonstrated with strains expressing 20 different PorA serosubtypes to protect against approximately 80% of sporadic MenB disease-causing strains in the United States.^50^ Historically, OMV vaccines have contained 1 PorA and have not demonstrated protection against strains with PorA sequences that are heterologous in amino acid sequence compared with the vaccine antigen.^51^ Therefore, selection of representative test strains to demonstrate that vaccine-elicited antibodies can be effective against a meningococcal disease strain is of paramount importance for protein-based vaccines.

Immune sera elicited by MenB-FHbp in preclinical and early clinical studies demonstrated broad bactericidal antibodies that could kill diverse MenB strains containing FHbp subfamily A and B variants heterologous to the vaccine FHbp variants A05 and B01.^35,52,53^ In an early assessment of the potential breadth of MenB-FHbp coverage, 100 MenB isolates with diverse FHbp variants, geographic origins, and genetic backgrounds were tested in hSBAs using MenB-FHbp immune rabbit serum.^27,35^ Of the 100 strains tested, 87 were killed in these hSBAs.^35^ Analysis of the 13 strains that were not killed suggested that the FHbp surface expression level on a given MenB strain affected the hSBA response. A threshold FHbp surface expression level was subsequently determined, above which isolates were predictably killed in hSBA.^54^ Additional investigations of potential factors determining strain susceptibility found that killing was largely independent of FHbp sequence variant, multilocus sequence type, or PorA subtype.^27,35^

To select strains with broad antigenic and epidemiologic diversity for clinical testing, over 1200 invasive MenB disease isolates were collected from laboratories and health agencies in the United States and Europe to represent the prevalence of MenB isolates that were contemporary at the time of collection; all strains contained the FHbp gene.^33^ An unbiased approach was used to select four antigenically and epidemiologically diverse representative test strains for use in MenB-FHbp immunogenicity studies.^27^ Selection criteria included expression of FHbp variants heterologous to the vaccine antigens and adequately reflecting the diversity of FHbp in MenB disease isolates, low to medium FHbp surface expression levels, and low baseline hSBA seropositivity rates. These four primary MenB test strains express FHbp variants from both FHbp subfamilies (strain [variant]: PMB2001 [A22], PMB80 [A56], PMB2707 [B24], and PMB2948 [B44]; Fig. 1).^32^

To supplement immunogenicity data generated using the four primary MenB test strains and to demonstrate that immune responses against the four primary MenB test strains are predictive of immune responses against the diversity of FHbp variants expressed by MenB disease-causing isolates, hSBAs using 10 additional test strains were developed. The 10 additional test strains were selected to include prevalent FHbp variants found in MenB disease-causing strains in the United States and Europe. Here, we (i) describe the strategy and criteria used to select the 10 additional test strains, and (ii) present data demonstrating that the immune responses measured by hSBA using the four primary MenB strains are predictive of the responses obtained using 10 additional test strains, which further demonstrate and support the broad coverage of the immune response elicited by MenB-FHbp.

## Results

### Sources and selection criteria for the additional MenB test strains

Nine of the 10 additional MenB test strains were obtained from a collection of 1263 invasive disease-causing MenB strains (the MenB isolate collection).^27,33^ For the MenB isolate collection, US strains were from the Active Bacterial Core Surveillance sites (2000–2005), covering ~13% of the population. European isolates (2001–2006) were from the public health laboratories of Norway, France, Czech Republic, and the Health Protection Agency in Manchester (which covers England, Wales, and Northern Ireland) and were collected systematically (every seventh or eighth isolate was included by order received at the country’s reference laboratory) and represented ~13% of invasive MenB isolates during the period.^33,55^ The strains expressing FHbp variant A07 were obtained from an extension of the MenB isolate collection that included an additional 551 disease-causing MenB strains from Spain and Germany (*n* = 1814). The extended MenB isolate collection was used as A07-expressing strains in the MenB isolate collection were not suitable because of the low surface expression of FHbp on these strains, high baseline seropositivity, and lack of readily available source of complement.

**Fig. 2:**
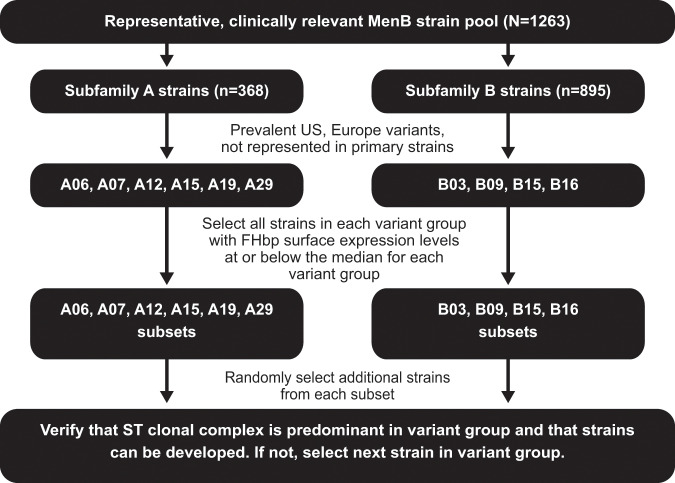
Algorithm used for the selection of additional *Neisseria meningitidis* serogroup B (MenB) test strains. The MenB serum bactericidal assay (SBA) isolate collection (*n* = 1263) is used as the example in this figure. FHbp (factor H binding protein), ST (sequence type).

The criteria used to select the additional MenB test strains were (i) FHbp variant prevalence among MenB disease-causing strains in the United States and/or Europe, (ii) the FHbp variant needed to be different from those expressed by MenB primary test strains, (iii) in vitro FHbp expression levels at or below median levels for the respective FHbp variant group to ensure that the strain was representative of the variant group it belonged to, (iv) technical compatibility in the hSBA, and (v) being considered a predominant clonal complex for the variant group (if a predominant complex existed). Strains meeting these criteria also needed to be technically compatible in the hSBA, including adequate availability of suitable human complement lots (Fig. 2). Strains in each FHbp variant group with expression levels below the cutoff level (i.e., at or below median levels for the respective FHbp variant group) were randomly selected, with the first strains within an FHbp variant group meeting the required genetic, phenotypic, and hSBA development criteria becoming the additional MenB test strains. An exception to this methodology was made for the strain expressing FHbp variant B03, which was selected in collaboration with, and using guidance provided by, the US FDA based on its previous use in a phase 2 study.^52^

### Characteristics of the additional MenB test strains

The 10 additional selected MenB test strains express FHbp variants A06, A07, A12, A15, A19, A29, B03, B09, B15, and B16 which differ from the ones in the four primary test strains (A22, A56, B24, B44) and have different sequences compared to the vaccine antigens (Table 1). The specific variants expressed by the four primary test strains are present in 42.0% (530/1263) of disease-causing isolates in the MenB isolate collection, and the specific variants expressed by the 10 additional test strains are present in an additional, non-overlapping 38.8% (490/1263) of disease-causing isolates in the MenB isolate collection (Fig. 1b).

**Table 1:** Characteristics of the 4 Primary and 10 Additional MenB Test Strains.

Strain	FHbp variant	Percentage identity to vaccine component	Strain MEASURE MFI^a^ (±1 SD)	FHbp variant group MEASURE MFI median^b^ (±1 SD)	Clonal complex	Country of isolation
Primary Strains						
PMB80	A22	88.9	3127 (2440, 4007)	2502 (1952, 3207)	CC41/44	United States
PMB2001	A56	98.1	5002 (3903, 6410)	5002^c^	CC213	France
PMB2948	B24	86.2	6967 (5436, 8929)	8457 (6599, 10,839)	CC32	France
PMB2707	B44	91.6	11,283 (8804, 14,461)	14,753 (11,511, 18,907)	CC269	United Kingdom
Additional Strains						
PMB3010	A06	96.2	3370 (2629, 4319)	3088 (2410, 3958)	CC461	United Kingdom
PMB3040	A07	85.4	1379 (1076, 1767)	1100 (858, 1409)	CC162	Germany
PMB824	A12	85.4	2540 (1982, 3255)	2467 (1925, 3161)	CC35	United States
PMB1672	A15	85.1	2995 (2337, 3838)	2904 (2266, 3721)	CC103	France
PMB1989	A19	88.1	1934 (1509, 2479)	1759 (1372, 2254)	CC8	United Kingdom
PMB3175	A29	93.1	3839 (2995, 4920)	5994 (4677, 7682)	CC32	United States
PMB1256	B03	90.8	3976 (3102, 5096)	2935 (2290, 3762)	CC41/44	United Kingdom
PMB866	B09	88.1	2089 (1630, 2677)	2275 (1775, 2916)	CC269	United Kingdom
PMB431	B15	86.5	3785 (2953, 4851)	4822 (3763, 6180)	CC41/44	United States
PMB648	B16	86.2	2347 (1831, 3008)	1996 (1557, 2558)	CC41/44	United Kingdom

*FHbp* factor H binding protein, *MenB* *Neisseria meningitidis* serogroup B, *MFI* mean fluorescence intensity, *SBA* serum bactericidal assay

^a^MFI ± 1 SD from MEASURE assay

^b^Based on the MenB SBA isolate collection (*n* = 1263), except for variant group A07, which was calculated from the extended MenB SBA isolate collection (*n* = 1814). Strains in each FHbp variant group with expression levels at or below median levels for the respective FHbp variant group were randomly selected. The cutoff level adopted for each FHbp variant group was the observed median MFI plus 1 SD, using the precision estimate of 25.2% relative SD

^c^There is only one strain expressing A56; thus, no SD values are included

### Immunogenicity analysis: subjects with hSBA titer ≥LLOQ for the 10 additional strains

The four primary strains were used to assess serological responses after two or three doses of MenB-FHbp in subjects participating in two pivotal phase 3 studies in adolescents and young adults.^32^ Serological responses to the 10 additional hSBA strains were assessed in a subgroup of the study subjects. The majority of subjects had hSBAs ≥ lower limit of quantitation (LLOQ; i.e., hSBA titer equal to 1:8 or 1:16, depending on strain) 1 month after dose 2 and 1 month after dose 3 for each of the primary (64.0–99.1% and 87.1–99.5%, respectively) and the 10 additional MenB test strains (51.6–100.0% and 71.3–99.3%, respectively) (Table 2). For the primary and additional MenB test strains, a substantial increase from baseline in the proportion of subjects achieving an hSBA titer ≥LLOQ was observed among MenB-FHbp recipients (0, 2, 6 month schedule) after the second MenB-FHbp dose, with additional increases after the third dose.

**Table 2:** Subjects with hSBA Titers ≥ LLOQ (1:8 or 1:16) for Primary and Additional MenB Test Strains^32^

FHbp Variant	% (95% CI) [*n*]					
	Adolescents^a^			Young adults^a^		
	Prevaccination	1 month after dose 2	1 month after dose 3	Prevaccination	1 month after dose 2	1 month after dose 3
Primary strains						
A22	33.2 (30.6, 35.9) [1238]	94.3 (92.9, 95.5) [1263]	97.8 (96.8, 98.5) [1266]	33.6 (31.3, 35.9) [1704]	84.7 (82.9, 86.4) [1697]	93.5 (92.2, 94.6) [1714]
A56	27.5 (24.9, 30.2) [1135]	99.1 (98.4, 99.5) [1222]	99.5 (98.9, 99.8) [1229]	32.2 (29.9, 34.5) [1657]	97.4 (96.5, 98.1) [1701]	99.4 (98.9, 99.7) [1708]
B24	6.4 (5.1, 7.9) [1264]	66.4 (63.6, 69.0) [1216]	87.1 (85.1, 88.9) [1250]	33.1 (30.9, 35.4) [1696]	86.5 (84.7, 88.1) [1685]	95.1 (93.9, 96.0) [1702]
B44	3.6 (2.6, 4.8) [1230]	64.0 (61.3, 66.8) [1204]	89.3 (87.4, 90.9) [1210]	11.0 (9.6, 12.6) [1716]	68.3 (66.1, 70.6) [1693]	87.4 (85.8, 89.0) [1703]
Additional strains						
A06	9.4 (6.2, 13.5) [277]	84.0 (75.0, 90.8) [79]	95.7 (92.6, 97.8) [280]	16.0 (11.9, 20.9) [275]	77.8 (67.8, 85.9) [90]	92.0 (88.1, 94.9) [275]
A07	43.1 (37.1, 49.3) [269]	93.8 (86.9, 97.7) [90]	96.4 (93.5, 98.3) [280]	55.8 (49.7, 61.8) [274]	97.9 (92.6, 99.7) [95]	95.7 (92.6, 97.7) [277]
A12	3.9 (2.0, 6.9) [280]	67.4 (57.0, 76.6) [64]	75.1 (69.6, 80.1) [277]	5.0 (2.8, 8.3) [278]	57.6 (46.9, 67.9) [92]	71.3 (65.5, 76.5) [275]
A15	20.7 (16.1, 26.1) [270]	65.6 (55.0, 75.1) [61]	87.2 (82.6, 91.0) [266]	37.3 (31.6, 43.2) [279]	83.2 (74.1, 90.1) [95]	91.8 (87.9, 94.7) [279]
A19	11.3 (7.8, 15.7) [274]	84.5 (75.8, 91.1) [82]	92.7 (89.0, 95.5) [275]	28.8 (23.5, 34.5) [278]	87.4 (79.0, 93.3) [95]	95.8 (92.7, 97.8) [284]
A29	17.5 (13.1, 22.5) [269]	100.0 (96.3, 100.0) [97]	98.6 (96.4, 99.6) [278]	31.1 (25.7, 36.9) [280]	96.8 (91.0, 99.3) [95]	99.3 (97.5, 99.9) [283]
B03	4.3 (2.2, 7.4) [280]	61.1 (50.3, 71.2) [55]	92.5 (88.7, 95.3) [279]	11.2 (7.7, 15.5) [277]	57.9 (47.3, 68.0) [95]	86.4 (81.8, 90.3) [273]
B09	15.2 (11.2, 19.9) [277]	76.3 (66.4, 84.5) [71]	86.2 (81.6, 90.1) [276]	23.5 (18.6, 28.9) [277]	65.3 (54.8, 74.7) [95]	77.0 (71.6, 81.9) [274]
B15	28.7 (23.5, 34.5) [275]	96.8 (90.9, 99.3) [90]	98.2 (95.9, 99.4) [281]	43.8 (37.8, 49.9) [274]	86.5 (78.0, 92.6) [96]	96.7 (93.9, 98.5) [276]
B16	7.6 (4.8, 11.4) [276]	61.6 (50.5, 71.9) [53]	81.7 (76.6, 86.0) [278]	21.9 (17.1, 27.3) [270]	51.6 (41.1, 62.0) [95]	78.0 (72.6, 82.8) [273]

Observed proportions of subjects were summarized with exact 2-sided 95% CIs using the Clopper-Pearson method. LLOQ = 1:16 for A06, A12, A19, and A22; LLOQ = 1:8 for A07, A15, A29, A56, B03, B09, B15, B16, B24, and B44

*FHbp* factor H binding protein, *hSBA* serum bactericidal assay using human complement, *LLOQ* lower limit of quantitation, *MenB* *Neisseria meningitidis* serogroup B

^a^Evaluable immunogenicity population

### Positive predictive values for the primary and additional strains

The relationship between vaccine-induced hSBA responses for the primary MenB test strains and the 10 additional MenB test strains was assessed (Table 3). Within an FHbp subfamily, positive predictive values (PPVs) were greater than 80% for most primary/additional strain pairs 1 month after dose 3. Thus, the immune responses measured in hSBAs using the primary test strains were highly predictive of immune responses for the additional strains within the same subfamily. The PPVs 1 month after dose 2 usually were slightly lower than those observed 1 month after dose 3 and ranged from 61.6% to 100% and 70.0% to 100% for subfamily A and B strain pairs, respectively, across studies. In summary, all PPVs showed high predictability for protective responses when comparing the primary and additional strain hSBA responses.

**Table 3:** Positive Predictive Value of Immune Response to Primary Strain for Immune Response to Additional Strain following MenB-FHbp Vaccination^32^

		% (95% CI)^a^ [*n/N*]b			
FHbp variant		Adolescents		Young adults	
Primary test strain	Additional test strain	1 month after dose 2	1 month after dose 3	1 month after dose 2	1 month after dose 3
A22	A06	89.7 (81.27, 95.16) [78/87]	96.0 (92.90, 97.97) [262/273]	87.5 (77.59, 94.12) [63/72]	94.0 (90.26, 96.59) [234/249]
	A07	98.9 (93.83, 99.97) [87/88]	96.3 (93.37, 98.23) [263/273]	100.0 (95.20, 100.00) [75/75]	99.2 (97.15, 99.90) [249/251]
	A12	72.7 (62.19, 81.68) [64/88]	75.9 (70.37, 80.90) [205/270]	67.6 (55.68, 78.00) [50/74]	77.9 (72.24, 82.91) [194/249]
	A15	70.9 (60.14, 80.22) [61/86]	89.7 (85.31, 93.18) [227/253]	92.4 (84.20, 97.16) [73/79]	93.9 (90.27, 96.47) [246/262]
	A19	87.8 (79.18, 93.74) [79/90]	95.4 (92.11, 97.60) [249/261]	97.5 (91.15, 99.69) [77/79]	98.9 (96.76, 99.77) [265/268]
	A29	100.0 (95.98, 100.00) [90/90]	99.6 (97.91, 99.99) [263/264]	98.7 (93.15, 99.97) [78/79]	100.0 (98.62, 100.00) [266/266]
A56	A06	84.3 (75.02, 91.12) [75/89]	96.3 (93.29, 98.21) [260/270]	83.3 (73.62, 90.58) [70/84]	93.0 (89.23, 95.71) [251/270]
	A07	94.4 (87.37, 98.15) [84/89]	97.0 (94.22, 98.71) [261/269]	98.9 (93.90, 99.97) [88/89]	96.0 (92.88, 97.96) [261/272]
	A12	68.2 (57.39, 77.71) [60/88]	75.6 (69.94, 80.61) [201/266]	61.6 (50.51, 71.92) [53/86]	72.2 (66.47, 77.48) [195/270]
	A15	64.4 (53.38, 74.35) [56/87]	89.2 (84.68, 92.76) [223/250]	84.6 (75.54, 91.33) [77/91]	92.0 (88.10, 94.90) [252/274]
	A19	83.5 (74.27, 90.47) [76/91]	93.8 (90.12, 96.41) [242/258]	90.1 (82.05, 95.38) [82/91]	96.4 (93.48, 98.26) [268/278]
	A29	100.0 (96.03, 100.00) [91/91]	98.9 (96.68, 99.76) [258/261]	97.8 (92.29, 99.73) [89/91]	99.6 (98.01, 99.99) [276/277]
B24	B03	80.3 (68.16, 89.40) [49/61]	97.1 (94.16, 98.83) [236/243]	75.7 (63.99, 85.17) [53/70]	89.9 (85.53, 93.28) [231/257]
	B09	88.7 (78.11, 95.34) [55/62]	92.1 (87.96, 95.19) [222/241]	82.9 (71.97, 90.82) [58/70]	80.5 (75.17, 85.20) [207/257]
	B15	100.0 (94.22, 100.00) [62/62]	99.6 (97.75, 99.99) [244/245]	100.0 (94.87, 100.00) [70/70]	98.8 (96.67, 99.76) [257/260]
	B16	82.1 (69.60, 91.09) [46/56]	86.4 (81.46, 90.46) [210/243]	70.0 (57.87, 80.38) [49/70]	81.3 (76.01, 85.90) [209/257]
B44	B03	78.9 (66.11, 88.62) [45/57]	96.6 (93.40, 98.52) [227/235]	88.9 (77.37, 95.81) [48/54]	95.8 (92.38, 97.96) [227/237]
	B09	88.3 (77.43, 95.18) [53/60]	90.1 (85.50, 93.61) [209/232]	96.4 (87.47, 99.56) [53/55]	85.9 (80.77, 90.09) [201/234]
	B15	100.0 (94.04, 100.00) [60/60]	99.2 (96.99, 99.90) [235/237]	100.0 (93.51, 100.00) [55/55]	98.3 (95.74, 99.54) [233/237]
	B16	84.9 (72.41, 93.25) [45/53]	85.5 (80.37, 89.77) [201/235]	79.6 (66.47, 89.37) [43/54]	83.8 (78.40, 88.24) [196/234]

LLOQ = 1:8 for strains expressing variants A07, A15, A29, A56, B03, B09, B15, B16, B24, and B44; LLOQ = 1:16 for strains expressing variants A06, A12, A19, and A22

*hSBA* serum bactericidal assay using human complement, *LLOQ* lower limit of quantitation, *MenB* *Neisseria meningitidis* serogroup B

^a^Exact 2-sided CI based on the observed proportion of subjects using the Clopper-Pearson method

^b^*N* = number of subjects with valid and determinate assay results for both the primary and additional strains with observed hSBA titer ≥ LLOQ for the primary strain at 1 month after vaccination 2 and at 1 month after vaccination 3; *n* = number of subjects with observed hSBA titer ≥ LLOQ for the given additional strain at 1 month after vaccination 2 and at 1 month after vaccination 3

## Discussion

A critical component of the clinical evaluation of the MenB-FHbp vaccine to determine the breadth of protection was the development of hSBAs using test strains with surface protein antigens whose sequence and expression variability are representative of the diversity of MenB disease-causing strains that were contemporary at the time of collection. As described in phase 3 studies in adolescents and young adults, hSBA response data for the four primary MenB test strains, all of which express FHbp variants heterologous to the vaccine antigens, strongly suggest that the bivalent MenB-FHbp vaccine provides broad coverage across diverse, disease-causing meningococcal strains. The 10 additional MenB test strains described here provide supportive immunologic data for MenB-FHbp and further confirm the validity of the use of the four primary test strains to measure the immune response to MenB-FHbp. As the responses obtained for the four primary test strains are predictive of the responses obtained for the additional 10 test strains, the immunological responses obtained by assessing the primary strains in hSBAs are representative of the diversity of strains causing invasive MenB disease.

For the hypothesis test-driven immunogenicity evaluations in licensure studies for MenB-FHbp, an unbiased approach was used to select the four primary MenB test strains from panels of disease-causing MenB collected in the United States and Europe. A similar method was used to select the 10 additional MenB hSBA test strains, taking into consideration specific selection criteria to ensure that test strains were representative of the antigenic diversity of MenB isolates.

Collectively, the 14 MenB test strains represent the majority of the prevalent meningococcal FHbp, with FHbp variants corresponding to ~80% of circulating invasive disease-causing isolates in the United States and Europe.

Positive predictive value analyses were used to determine the association of immune responses, measured by hSBA, among primary and additional test strains expressing FHbps within the same subfamily. All of the PPV analyses showed the high predictability of the protective responses against the primary strain for the protective responses observed against the additional strains. These PPV analyses indicate that the responses observed against the four primary MenB test strains are representative of responses to other disease-causing MenB strains that express additional sequence-diverse FHbp variants different from the vaccine antigen variants.

The MenB-FHbp–elicited responses measured by hSBA to the four primary and 10 additional MenB test strains were evaluated using sera from individual vaccine recipients. By determining the proportion of vaccinated subjects with functional bactericidal antibodies, assessment of the breadth of MenB-FHbp coverage at the individual level was determined, which is not possible using pooled sera. The four primary MenB test strains were selected to represent the diversity of MenB disease-causing IMD and thus support the potential breadth of coverage for MenB-FHbp using hSBA.^47^ Responses of individuals with hSBA titers ≥1:4 are the accepted correlate of protection and a surrogate of meningococcal vaccine efficacy.^41–43,56^ Thus, the responses provide a comprehensive and biologically predictive assessment of breadth of vaccine coverage. The relevance of the hSBA responses to the four primary MenB test strains to describe breadth of vaccine coverage is supported by the demonstration of protective bactericidal responses by MenB-FHbp also observed against diverse and contemporary MenB outbreak strains from Europe and the United States^47,57^ and against non-MenB disease-causing strains (ie, meningococcal serogroups C, Y, W, and X).^58^

Another methodology, the enzyme-linked immunosorbent assay–based Meningococcal Antigen Typing System (MATS), has been used to predict vaccine coverage of MenB-4C.^59,60^ However, MATS only predicts coverage of antigens specific to MenB-4C and is not useful for assessing coverage of other vaccines with different antigen compositions.^47,61^ Specifically, MATS measures antigen expression rather than bactericidal activity and is reported as a relative potency compared with a reference strain for each antigen.^61^ If the relative potency for any one of the component antigens is commensurate with bactericidal activity for MenB-4C immune sera (i.e., achieves a positive bactericidal threshold), the strain is considered susceptible to killing.^62^ However, because sera from vaccinated individuals are not used in MATS,^61^ the assay is unable to predict the proportion of a population achieving hSBA titers ≥1:4 (i.e., the correlate of protection) in response to immunization.

Of note, limitations in performing hSBAs exist.^47^ For example, hSBAs are labor intensive and can require large quantities of sera and assay-compatible complement, particularly when larger numbers of strains and/or sera are to be assessed. In addition, interlaboratory differences in the performance of the assay reagents and strains used in hSBAs limit comparison of responses and assessments of breadth of coverage between vaccines. A known limitation of PPV analysis is the dependence of the magnitude of the response on prevalence (i.e., in this setting, the proportion of subjects achieving hSBA ≥LLOQ for the additional strains).^63^ However, it is notable in this analysis that although there was a range of postvaccination responses to the additional strains (at 1 month postdose 2 and postdose 3), PPVs were uniformly high.

Taken together, the immunogenicity data obtained from the 10 additional MenB hSBA test strains support the response data obtained from the four primary MenB hSBA test strains and confirm the broad coverage of MenB isolates conferred by MenB-FHbp. This work demonstrates the application of a rigorous assessment of a MenB vaccine’s elicited immune response using the epidemiology of MenB strains with regard to the vaccine antigen sequence and expression, in conjunction with the recognized surrogate of protection (hSBA), and, using this knowledge, led to vaccine licensure.

## Methods

### Quantitation of FHbp surface expression

For all strains, FHbp surface expression was quantified by the validated MEASURE assay, a flow cytometric assay using monoclonal antibody (MN86–994–11) recognition of a conserved FHbp epitope common to both FHbp subfamilies.^27,35^ Details of the MEASURE assay have been described previously.^54^ Briefly, 50 µL of bacteria were plated per well of 96-well round-bottom polystyrene plates, then centrifuged and washed with 1% (weight/volume) bovine serum albumin (BSA) in 1X phosphate buffered saline (PBS). Monoclonal antibodies (MN86–994–11 [in-house antibody] or mouse IgG [negative control; Jackson Immunoresearch, Cat #015–000–003, West Grove, PA, USA]) were added to the bacterial pellets to a final concentration of 6.7 µg/mL (335 ng/well), resuspended, and incubated for 30 min on ice. Cells were washed twice in 1% BSA in PBS followed by addition of biotinylated goat anti-mouse IgG (subclasses 1 + 2a + 2b + 3; Jackson Immunoresearch, Cat #115–065–165; 10 µg/mL, 500 ng/well), resuspension, and incubation for 30 min on ice. After two washes, cells were resuspended in streptavidin-phycoerythrin (PE; BD Biosciences, Cat #554061, San Jose, CA, USA; 5 µg/mL, 250 ng/well). Cells were washed twice in 1% BSA in PBS and then resuspended in 1% paraformaldehyde. For each well, 20,000 events were acquired on an Accuri C6 flow cytometer and analyzed using Accuri CFlow software (BD Biosciences). The gating strategy of the MEASURE assay has been previously published.^54^ For each sample, the mean fluorescence intensity (MFI) of the PE channel was determined after gating in the logarithmic forward scatter versus side scatter dot plot. FHbp expression was considered above the level of detection if MFIs were above an arbitrary threshold of at least 100 and three times that of the negative control (mouse IgG) MFI used in that assay. The cutoff level adopted for each FHbp variant group was the observed median mean fluorescence intensity plus 1 standard deviation, using the precision estimate of 25.2% relative standard deviation.

### Immunogenicity analysis

Each of the 10 additional MenB test strains were used in hSBAs to test sera from subjects participating in 2 pivotal phase 3 studies of MenB-FHbp.^32^ A total of 900 subjects from each study were to be divided into three subsets (*n* = 300 each); the 10 additional test strains were allocated across these subsets so that two subsets each included 3 test strains and 1 subset included four test strains. The subsets included samples from 300 subjects to ensure that ≥150 evaluable hSBA results from each study would be obtained. Immune responses measured by hSBA using phase 3 clinical study sera were based on the assay LLOQ, which was an hSBA titer equal to 1:8 or 1:16 depending on the strain.

### Positive predictive value analyses

The PPV for each primary/additional strain pair within an FHbp subfamily was defined as the proportion of subjects responding to the additional strain (hSBA titer ≥LLOQ for the additional strain) among the total number of primary strain responders (hSBA titer ≥LLOQ for the primary strain). PPV analyses assessed whether observed hSBA responses to the four primary strains predicted immune responses to additional strains expressing FHbps from the same subfamily.

### Reporting summary

Further information on research design is available in the Nature Research Reporting Summary linked to this article.

## Supplementary information

Reporting Summary

## Data Availability

Upon request, and subject to certain criteria, conditions and exceptions (see https://www.pfizer.com/science/clinical-trials/trial-data-and-results for more information), Pfizer will provide access to individual de-identified participant data from Pfizer-sponsored global interventional clinical studies conducted for medicines, vaccines and medical devices (1) for indications that have been approved in the US and/or EU or (2) in programs that have been terminated (i.e., development for all indications has been discontinued). Pfizer will also consider requests for the protocol, data dictionary, and statistical analysis plan. Data may be requested from Pfizer trials 24 months after study completion. The de-identified participant data will be made available to researchers whose proposals meet the research criteria and other conditions, and for which an exception does not apply, via a secure portal. To gain access, data requestors must enter into a data access agreement with Pfizer.
